# Leukocytoclastic vasculitis with purpura and renal failure induced by the anti-epidermal growth factor receptor antibody panitumumab: a case report

**DOI:** 10.1186/s13256-018-1877-7

**Published:** 2019-01-16

**Authors:** Hitomi Kamo, Eiji Shinozaki, Takanobu Sugase, Nobuyuki Mizunuma, Shoji Taniguchi, Takashi Gotoh, Keisyo Chin, Tomoaki Tanaka, Kazumi Koga, Kensei Yamaguchi

**Affiliations:** 1 Department of Surgery, Koga General Hospital, Miyazaki, Japan; 2 Department of Gastroenterological Chemotherapy, The Cancer Institute Hospital, Jaese Foundation for Cancer Research, Tokyo, Japan

**Keywords:** Anti-EGFR antibody, Colon cancer, Renal toxicity, Purpura, Leukocytoclastic vasculitis, Panitumumab

## Abstract

**Background:**

Panitumumab is the first human combinatorial antibody for the treatment of metastatic colorectal carcinoma. Dermatologic toxicity of all grades occurs in more than 90% of patients. However, there are few reports of purpura induced by anti-epidermal growth factor receptor antibody. Renal failure is also uncommon as an adverse event of anti-epidermal growth factor receptor antibody.

**Case presentation:**

A 67-year-old Japanese man with advanced colon cancer received monotherapy with panitumumab. General malaise, bilateral edema of his legs, and bilateral purpura of his forearms developed 2 days after the second cycle of panitumumab. A skin biopsy was performed to evaluate the purpuric lesions on his left leg and leukocytoclastic vasculitis was diagnosed. Blood tests showed grade III acute renal failure with a blood urea nitrogen level of 33.8 mg/dL and a creatinine level of 3.10 mg/dL.

**Conclusions:**

This is the first reported case of leukocytoclastic vasculitis followed by purpura and acute renal failure associated with panitumumab.

## Background

Panitumumab is a fully humanized antibody for the treatment of *RAS* wild-type metastatic colorectal carcinoma (mCRC). Panitumumab monotherapy is generally well tolerated, and the major adverse effects are skin toxicities, including some severe events. Dermatologic toxicity of all grades occurs in more than 90% of patients [1]. However, there are few reports of purpura induced by anti-epidermal growth factor receptor (EGFR) antibody. Renal failure is also uncommon as an adverse event of anti-EGFR antibody. We describe a patient with advanced colon cancer with bilateral edema of the legs and bilateral purpura noted 2 days after a second cycle of panitumumab. Leukocytoclastic vasculitis (LCV) was diagnosed with a skin biopsy; blood tests showed grade III acute renal failure. This is the first reported case of LCV followed by purpura and acute renal failure associated with panitumumab.

## Case presentation

A 67-year-old Japanese man with advanced colon cancer with liver metastasis presented with bowel obstruction in May 2007 and underwent emergency surgery (left hemicolectomy with D3). A pathological examination revealed a well-to-moderately differentiated, type 2, intermediate-type tubular adenocarcinoma (70 × 40 mm) arising in the descending colon. The lesion was associated with pathological evidence of serosal invasion (pSE), an infiltrative growth pattern (INFβ), moderate lymphatic invasion (ly2), and moderate venous invasion (v2). There was no involvement of the proximal margin (pPM0, 150 mm), no distant metastasis (pDM0, 120 mm), and no lymph node metastasis (0/27). A liver biopsy revealed metastatic adenocarcinoma.

His medical history indicated a gastric ulcer in 2003. We did not note any personal or family history of kidney disease, autoimmune disease, or asthma. He worked in an office. He had smoked five cigarettes per day for 50 years and drank alcohol socially.

**Fig. 1:**
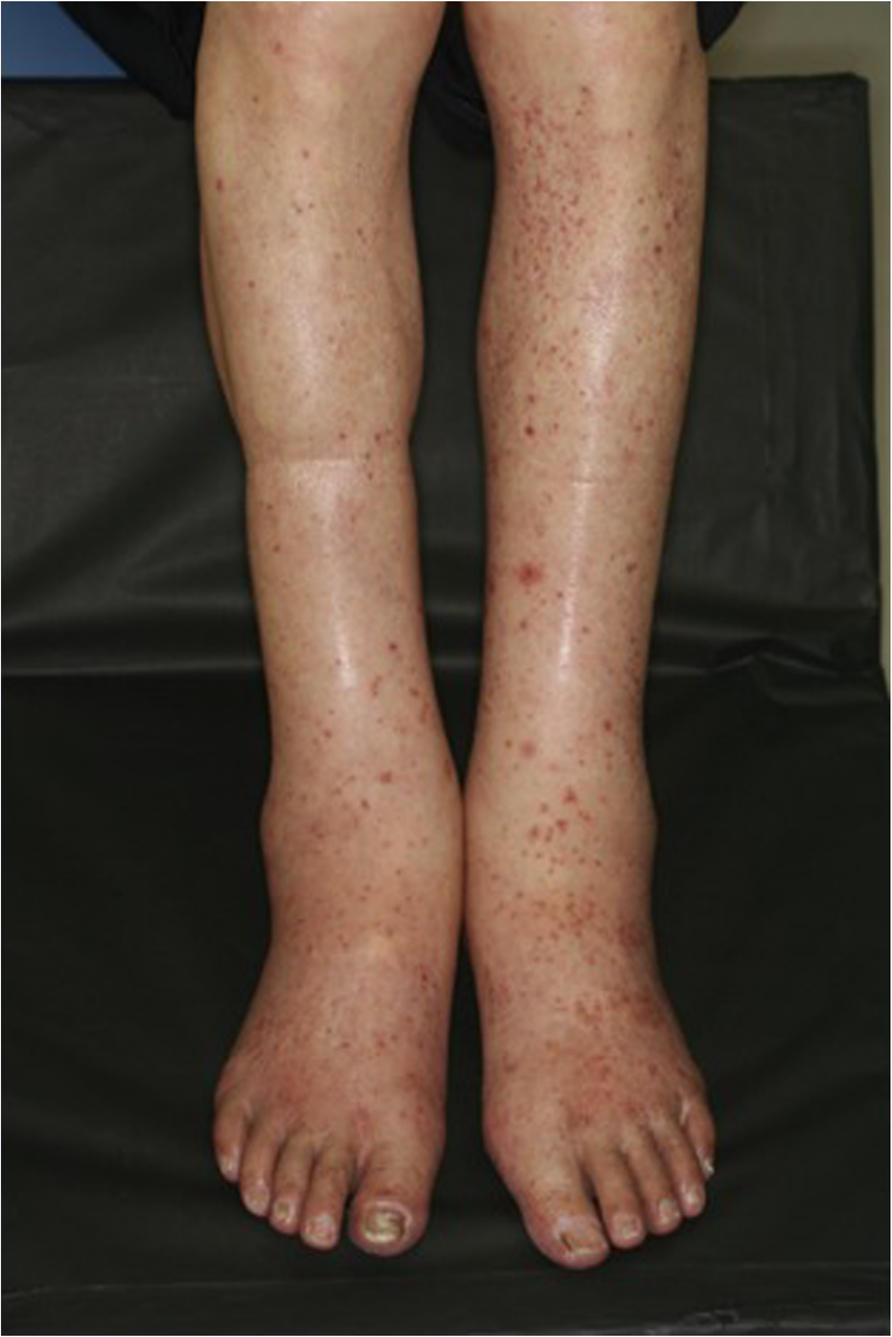
Bilateral edema of the legs

**Fig. 2:**
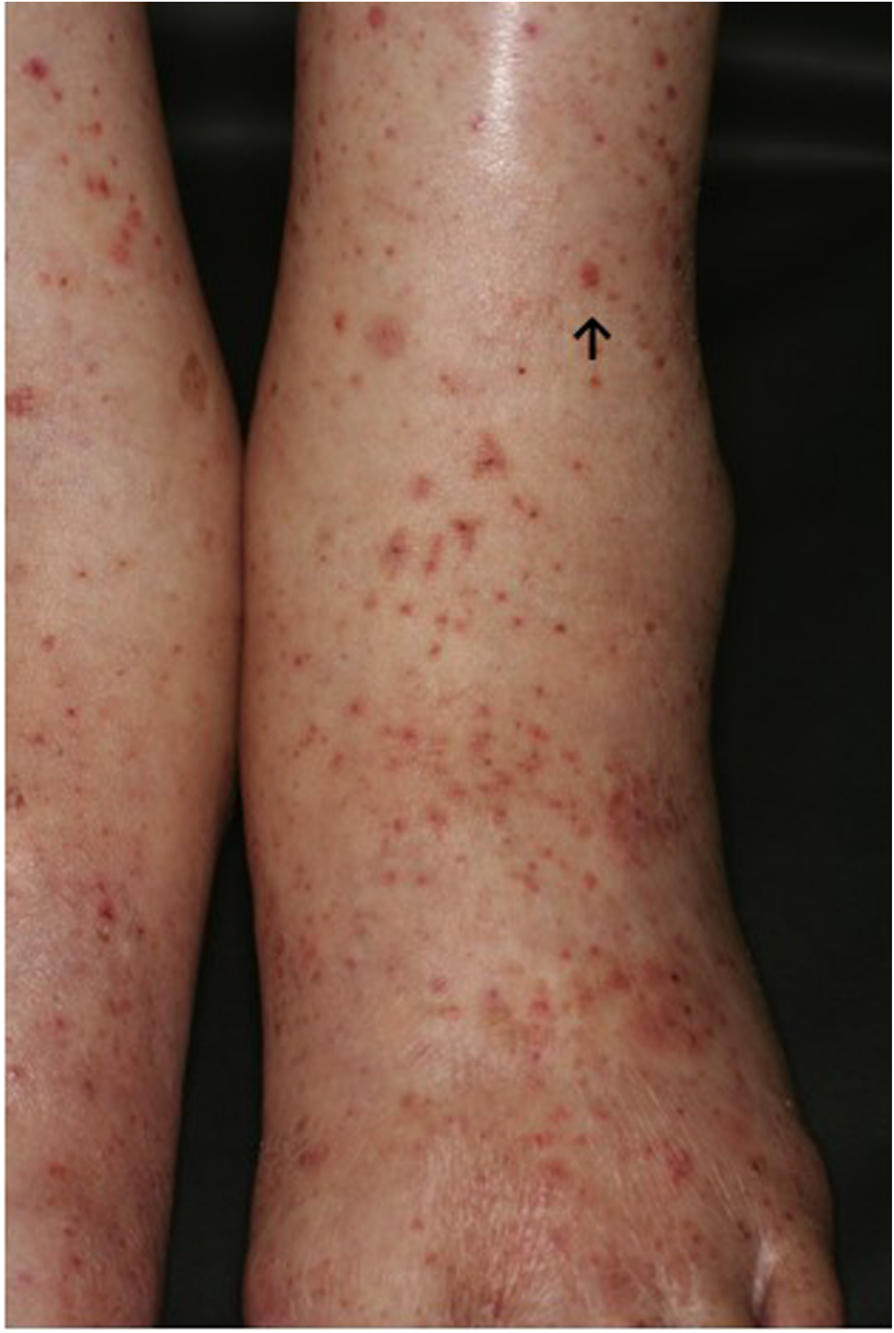
Bilateral palpable purpura of the forearms was noted. ↑Skin biopsy of this lesion was performed

One month after the operation, he initially received hepatic arterial infusion therapy with 5-fluorouracil (5-FU) from June through to October 2007. After receiving five courses of simplified l-leucovorin plus 5-FU (sLVFU), he had strangulating intestinal obstruction and underwent emergency surgery in January 2008. Second-line treatment with fluorouracil, leucovorin, and irinotecan (FOLFIRI) was started in October 2008 and terminated in May 2009 as a result of renewed progression. From June 2009 he received third-line treatment with modified leucovorin, fluorouracil, and oxaliplatin regimen (mFOLFOX-6) plus bevacizumab. However, in June 2010 a computed tomography (CT) scan revealed progression of liver metastasis again. Considering that our patient had already been treated with the combination chemotherapies FOLFIRI and mFOLFOX-6 and the wild-type *RAS* status of his primary tumor, treatment with bi-weekly panitumumab monotherapy (500 mg/m^2^) was initiated on July 20, 2010. He had no adverse events after the initial course of panitumumab. A second course of panitumumab was administered on August 2, 2010. General malaise, leg swelling, and skin rash developed 2 days after the second cycle of panitumumab (2 weeks after the initial dose), and around August 18 the symptoms intensified. However, he had neither joint pain nor abdominal pain during the period. When he visited the out-patient department on August 23, bilateral edema of his legs and bilateral purpura of his forearms had progressed (Figs. 1 and 2). Blood tests showed grade III acute renal failure with blood urea nitrogen (BUN) level of 33.8 mg/dL and a creatinine level of 3.10 mg/dL, as well as nephrotic syndrome with a total protein (TP) level of 4.5 g/dL and an albumin level of 1.4 g/dL. Urine analysis showed blood (3+) and urinary protein (4+). Several acanthocytes and 5–9 white blood cell casts were observed in the urinary sediment. He was therefore immediately admitted to our hospital. His height was 164.cm and body weight was 50 kg (6 kg increase in 3 weeks). His blood pressure was 110/60 mmHg and pulse rate was 84 beats per minute. His body temperature was 36.4 °C. The results of his physical examination were relatively unremarkable, except pretibial pitting edema and diffuse purpura on his whole body. There was no neurologic abnormality including mononeuropathy multiplex.

**Fig. 3:**
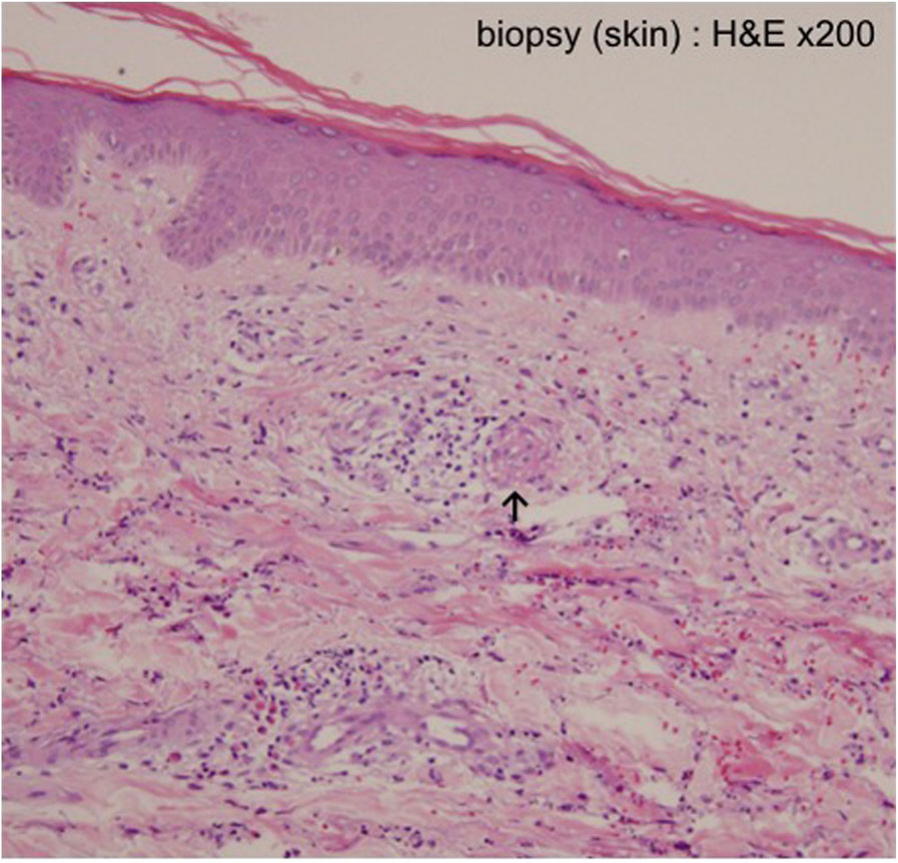
Dense infiltration of neutrophils and lymphocytes can be seen around the small vessels in the upper dermis. A large amount of nuclear debris is present. ↑ Fibrinoid necrosis is suspected

**Fig. 4:**
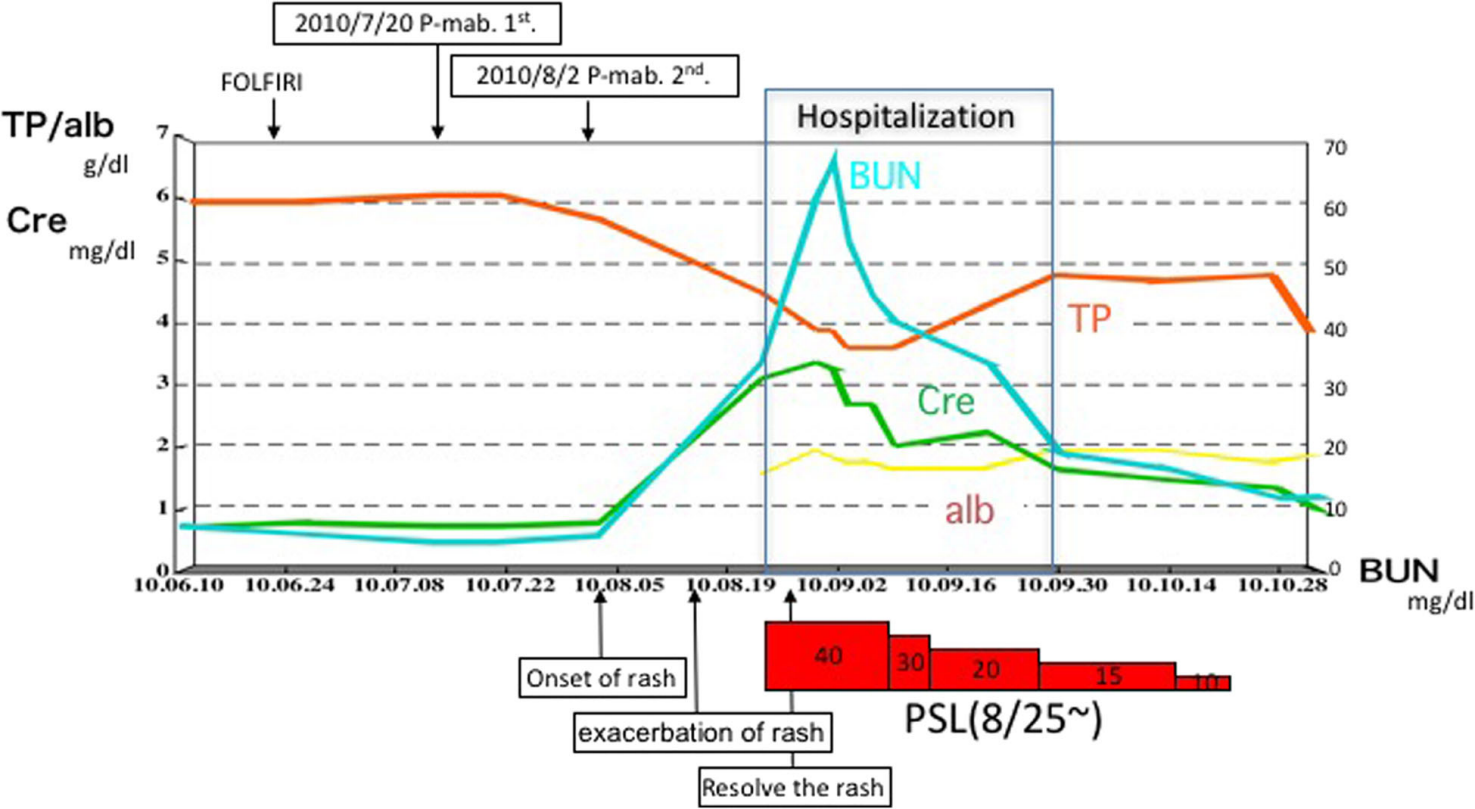
The treatment outcome after immediate hospitalization. The patient was discharged after 3 weeks. *alb* albumin, *BUN* blood urea nitrogen, *Cre* creatinine, *FOLFIRI* fluorouracil, leucovorin, and irinotecan, *P-mab* panitumumab, *PSL* prednisolone, *TP* total protein

He underwent examinations for differential diagnosis from other kidney diseases: immunoglobulin G (IgG), immunoglobulin A (IgA), immunoglobulin M (IgM), C3, C4, cryoglobulin, proteinase 3-antineutrophil cytoplasmic antibody (PR3-ANCA), and myeloperoxidase-antineutrophil cytoplasmic antibody (MPO-ANCA). However, no clinically significant findings were obtained (Table 1). Because oliguria (urine volume, 400 mL/day) was present after admission, an albumin preparation (12.5 g twice daily) and furosemide were administered for 3 days. Treatment with prednisolone 40 mg/day was begun immediately. After this treatment, his urine volume increased to 1100 mL, and the generalized edema improved slightly. A skin biopsy was performed to evaluate the purpuric lesions on the lateral lower region of his left leg on August 25, and LCV was diagnosed (Fig. 3). A drug lymphocyte stimulation test (DLST) was performed as a supplementary test to differentiate the cause of the drug-induced allergic symptoms. However, the results of all tests were negative for both cetuximab and panitumumab (Table 1). As local therapy, betamethasone ointment and moisturizer were applied topically. The skin lesions gradually improved, and only crust remained on August 31. Around August 27, his urine volume decreased to 600–900 mL/day, and edema and his body weight increased. Thus, treatment with indapamide was started on August 31. After this treatment, his urine volume increased to 1500–1700 mL/day. The urinary protein excretion decreased from 7.14 g/day to 6.83 g/day during hospitalization, indicating that he had nephrosis. His kidney function gradually improved after his BUN and creatinine reached peak levels of 60.5 mg/dL and 3.36 mg/dL, respectively, on August 30. His levels of BUN and creatinine on September 9 were respectively 40.1 mg/dL and 2.01 mg/dL, indicating a tendency to decrease, and he was discharged from our hospital on September 13 (Fig. 4).

**Table 1:** Blood examination and urinary test

Blood examination	Result	Normal value
lgG	646 mg/dl	870–1700
lgA	309 mg/dl	110–410
lgM	94 mg/dl	33–190
C3	77 mg/dl	86–160
C4	21 mg/dl	17–45
Cryoglobulin	Pseudo positive	negative
Antinuclear antibody (ANA)	< × 40	< × 40
Serum complement level	25 CH50/ml	25.0–48.0
PR3-ANCA	< 10 EU	< 10
Urine protein per day	11.77 g/day	< 0g/day
Urinary test		
Specific gravity 1.030, pH 6.5, protein (4+), uric blood (3+)		
Glucose (1+), ketone body (−), urobilinogen (±), leukocyte (±)		
DLST		
Cetuximab	negative	
Panitumumab	negative	

*DLST* drug lymphocyte stimulation test, *IgG* immunoglobulin G, *IgA* immunoglobulin A, *IgM* immunoglobulin M, *MPO-ANCA* myeloperoxidase-antineutrophil cytoplasmic antibody, *PR3-ANCA* proteinase 3-antineutrophil cytoplasmic antibody

**Fig. 5:**
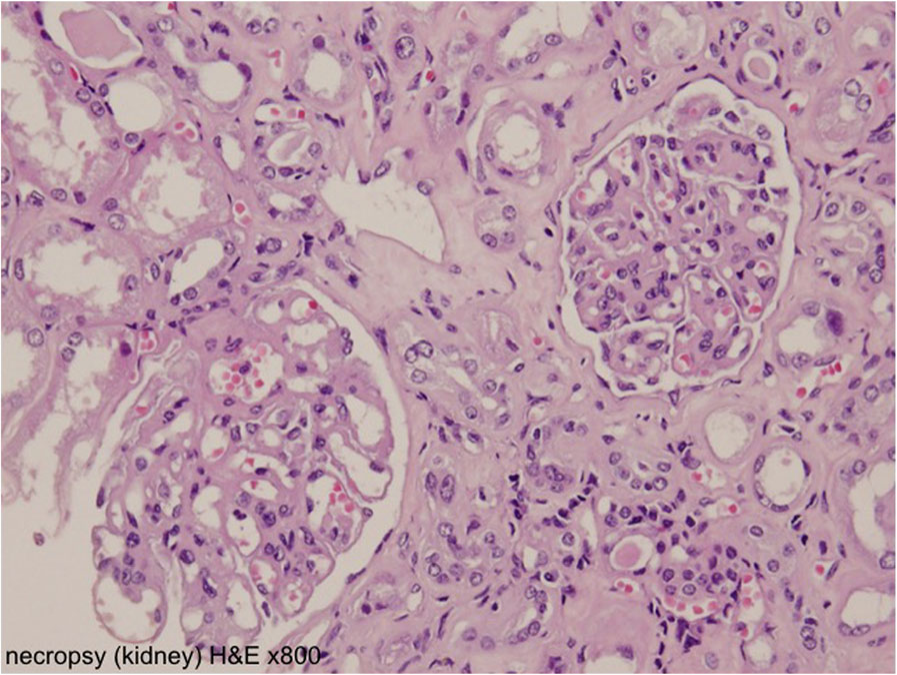
A needle necropsy of the kidney. Some glomeruli show mesangial matrix expansion with segmental mesangial hypercellularity (hematoxylin and eosin × 800)

**Fig. 6:**
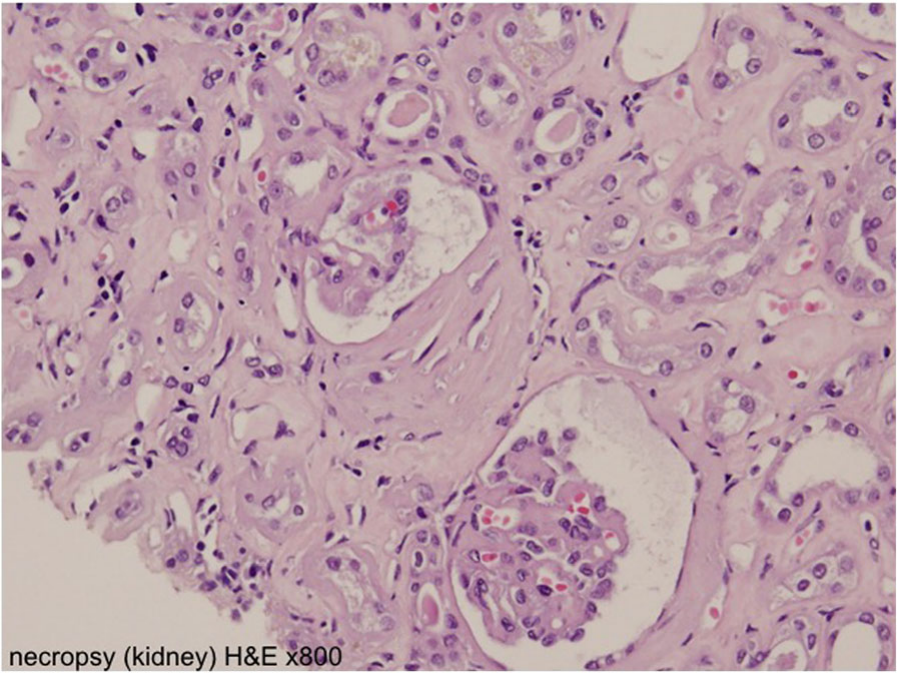
A needle necropsy of the kidney. Some glomeruli had collapsed (hematoxylin and eosin × 800)

Because the nephrotic syndrome continued, he was hospitalized for kidney biopsy on November 1, but it was cancelled due to emerging hydronephrosis. His serum magnesium level was 1.5 mg/dl (1.9–2.5 mg/dl). This case was discussed at a multidisciplinary conference of the Cancer Institute Hospital. Rechallenge of panitumumab was denied considering the increasing nephrotoxicity. The best supportive care was eventually provided. On November 11, our patient agreed with our decision to provide supportive care. He died of colon cancer progression in May 2011, 48 months after the onset of initial symptoms and after having received 9 months of best supportive care. A needle necropsy of the kidney was performed approximately 40 minutes after death. Global sclerosis was found in 6 of approximately 50 glomeruli, and fibrous crescent formation was recognized in 3 glomeruli, while the components of other glomeruli had collapsed (Figs. 5 and 6). On immunohistochemical staining, no deposition of IgA or IgG was found in the glomeruli.

## Discussion

This case is unusual because purpura is a rare form of skin toxicities of panitumumab, most skin toxicities are acne-like rash, cracking, and dryness. Also there are few reports of panitumumab-associated renal failure. Panitumumab is the fully humanized IgG2 monoclonal antibody for the treatment of *RAS* wild-type mCRC. In Japan, panitumumab was approved by the Ministry of Health, Labour and Welfare in April 2010 and was launched in June of that year. It has been used to treat colorectal cancer and head and neck cancer for more than 5 years. Panitumumab is generally well tolerated, and the major toxic effects are skin reactions, including some severe events. Skin reactions are ascribed to EGFR expressed on basal epidermal keratinocytes, sebaceous and eccrine sweat gland cells, and various cancer cells. Dermatologic toxicity of all grades occurs in more than 90% of patients; the management of skin reactions thus plays a very important role in continuing treatment [1]. The most common dermatologic reactions caused by anti-EGFR antibodies are acne-like rash, cracking, dryness, infection of the fingernails or toenails, itching, and redness, whereas purpura is rare. A skin biopsy of purpura showed LCV in our patient. LCV is a histopathological term commonly used to denote small-vessel vasculitis [2]. LCV is defined histologically as a predominantly neutrophilic perivascular infiltrate associated with cutaneous postcapillary venules with fibrinoid deposits in and around the vessel wall, endothelial swelling, leukocytoclasis, and extravasations of red blood cells. LCV may be associated with systemic involvement. Internal disease most often manifests in the joints, the gastrointestinal tract, and the kidneys. LCV may occur secondary to medications, underlying infection, collagen-vascular disorders, or malignancy. In our patient, general malaise, leg swelling, and skin rash developed 2 days after the second cycle of panitumumab. He had no signs of infection or collagen-vascular disorders. The classic symptoms of vasculitis are rash, joint pain and swelling, abdominal pain, and/or related kidney disease. Purpura and renal toxicity were severe but neither joint pain nor abdominal pain was observed in our patient.

There are several diseases that need to be differentiated from LCV in adults, such as allergic cutaneous vasculitis and cutaneous LCV, vasculitis associated with collagenosis, microscopic polyangiitis, Churg–Strauss syndrome, Wegener’s granulomatosis, septic vasculitis, and urticarial vasculitis. Although our patient underwent examinations for differential diagnosis from other kidney diseases (IgG, IgA, IgM, C3, C4, cryoglobulin, PR3-ANCA, and MPO-ANCA), no clinically significant findings were obtained. DLST was performed as a supplementary test to differentiate the cause of the drug-induced allergic symptoms. However, the results of all tests were negative for both cetuximab and panitumumab. On performing a literature search of PubMed for the period up to 2016 using the keywords LCV, purpura, and renal toxicity related to anti-EGFR antibody, we identified several cases of LCV associated with purpura. Two cases involved panitumumab (including our case) [3], one case involved cetuximab [4], five involved gefitinib [5–7], and three cases involved erlotinib [8, 9] (Table 2). The rash improved in all of these patients, and renal failure did not develop. Paraneoplastic vasculitis was suspected in two patients. Henoch–Schönlein purpura (HSP) induced by anti-EGFR antibody was strongly suspected in one patient because the onset was during treatment with anti-EGFR antibody, not before or after the initial diagnosis of malignant tumor. Nephrotoxicity might have been one of the symptoms of LCV in the patient. Other possible causes of nephrotoxicity are a direct adverse event of panitumumab, paraneoplastic syndrome, or another kidney disease occurring by chance. Only 12 (0.4%) cases of renal and urinary disorders induced by panitumumab were reported among 3085 Japanese patients participating in a post-marketing surveillance study [1]. In addition, one patient who received cetuximab monotherapy had diffuse proliferative glomerulonephritis [10], and another patient had nephrotic syndrome associated with cetuximab [11].

**Table 2:** Purpura and renal toxicity by anti-epidermal growth factor receptor antibody

Agent	Purpura (number of patients)	Renal toxicity (number of patients)	Purpura and renal toxicity (number of patients)
Panitumumab	2	2	1
Cetuximab	1	2	1
Gefitinib	5	4	0
Erlotinib	3	1	0

Although our patient underwent examinations for differential diagnosis from other kidney diseases associated with antibodies, no clinically significant findings were obtained. The possibility of postrenal renal failure or apparent infiltration into the kidney was ruled out by CT findings, and there was no blood clot in major blood vessels (that is, no evidence of disseminated intravascular coagulation). A needle necropsy of the kidney was performed approximately 40 minutes after death. No evidence of membranous nephropathy associated with malignant tumor or of purpura nephritis was found on biopsy.

On performing a literature search of PubMed using the keywords “renal toxicity caused by anti-EGFR antibody,” two cases involving panitumumab (including our case), one case involving cetuximab, four cases involving erlotinib, and one case involving gefitinib were found [12, 13]. Patients with both purpura and renal toxicity caused by anti-EGFR antibody are rather rare. Only one patient had both HSP and renal toxicity caused by cetuximab. No reported case had both LCV and renal toxicity caused by panitumumab.

This is the first reported case of LCV followed by the development of acute renal failure associated with panitumumab. This diagnosis was based on the facts that our patient had received panitumumab monotherapy, and the onset of symptoms was 2 to 3 weeks after the initial dose.

## Conclusions

This is the first reported case in which panitumumab-induced LCV was followed by purpura and the development of acute renal failure.

We learned that severe renal toxicities occur with purpura from the presented case. However, the use of anti-EGFR antibody is expected to increase in the future. Renal toxicity should therefore be carefully evaluated in patients in whom an unusual rash develops after the onset of treatment.

## Abbreviations

5-FU: 5-Fluorouracil
BUN: Blood urea nitrogen
CT: Computed tomography
DLST: Drug lymphocyte stimulation test
EGFR: Epidermal growth factor receptor
FOLFIRI: Fluorouracil, leucovorin, and irinotecan
HSP: Henoch–Schönlein purpura
LCV: Leukocytoclastic vasculitis
mCRC: Metastatic colorectal carcinoma
mFOLFOX-6: Modified leucovorin, fluorouracil, and oxaliplatin regimen
MPO-ANCA: Myeloperoxidase-antineutrophil cytoplasmic antibody
PR3-ANCA: Proteinase 3-antineutrophil cytoplasmic antibody
